# Supplementary material for: Management of acute acquired comitant esotropia using prisms and vision therapy
Source: PLoS One. 2026 Feb 23;21(2):e0340010. doi: 10.1371/journal.pone.0340010 (PMC12928452; doi:10.1371/journal.pone.0340010)
Supplement: S1 File — (DOCX) [file pone.0340010.s001.docx]

***Vision Therapy Protocol for improving divergence***

1. The first line of management is to make sure the patient has the appropriate refractive correction, confirmed after cycloplegic refraction.
2. Baseline phoria measures (distance and near), fusional vergence ranges (distance and near) needs to be measured before starting the therapy. The same needs to be measured after the therapy is completed.
3. Usually 10 sittings of in-office vision therapy is given – that includes training both vergence and accommodation.
4. In vergence training specific focus is given for divergence, and towards the last few sessions of the therapy both convergence and divergence is trained to ensure good flexibility is present in both directions
5. The battery of divergence exercises for in-office therapy includes double aperture ruler, vectogram, VTS4 divergence exercises given at different viewing distances (near and distance) and Brock string (also given as home exercise)
6. Accommodation exercises are also given such as Hart chart and accommodative flippers.
7. and Brock string. Patients were also encouraged to do the Brock string exercise at home. For those who could not come for in-office therapy only Brock string exercise was given for a minimum duration of 20 minutes every day, twice a day. The typical in-office vision therapy was given for a duration of 45-60 minutes in a session.

In Brock string, the patients were oriented to appreciate the physiological diplopia. They were asked to maintain the fixating bead single for 20 seconds and encouraged to move that bead farther away till the threshold for double is reached. At this break point, they were encouraged to see if they can make the bead single. When not possible, the bead was brought in a little closer to achieve single vision, and again from that distance, the patient was asked to take the bead back, maintaining clear, single vision. Patients were encouraged to monitor the limit break point distance and encouraged to increase that distance every day. With Brock string, vergence rock exercises were also combined, where the patients were asked to look at the 3 beads at different distances in the string, each time to hold the fixating bead clear and single for 20s, while appreciating physiological diplopia, and then to view the other beads doing the same. Such an exercise would improve the vergence facility to make eye movements without noticing the double vision and locking fusion seamlessly at different viewing distances.

For in-office therapy, usually 10 sittings that can be completed in 2-weeks or spread over few weeks (depending on patient’s ability to come) were given. A variety of exercises to improve vergence range (first focusing mostly on divergence and later with both convergence and divergence) were given. As accommodation and vergence are coupled mechanisms, therapy exercises also include accommodation and disaccommodation. Binocular vision parameters were measured before (baseline) and after the vision therapy sessions. 
