# Supplementary material for: Management of acute acquired comitant esotropia using prisms and vision therapy
Source: PLoS One. 2026 Feb 23;21(2):e0340010. doi: 10.1371/journal.pone.0340010 (PMC12928452; doi:10.1371/journal.pone.0340010)
Supplement: Attachment — Submitted filename: AACE_26Feb2025_ver2.docx [file pone.0340010.s002.docx]

**Management of Acute Acquired Comitant Esotropia Using Prisms and Vision Therapy**

PremNandhini Satgunam,^a,b^ Rinki Gupta,^a,c^ Manali Sikder, ^a,c^ Rohan Nalawade,^c^
Ramesh Kekunnaya^c^

**Short title:** Management of AACE

**Author Affiliations:**

^a^ Brien Holden Institute of Optometry and Vision Science

^b^ Hyderabad Eye Research Foundation, L V Prasad Eye Institute, Hyderabad, India

^c^ Child Sight Institute, Jasti V Ramanamma Children’s Eye Care Center, L V Prasad Eye Institute, Hyderabad, India

**Corresponding Author:**

PremNandhini Satgunam, BS Opt, MS, PhD

Hyderabad Eye Research Foundation

L V Prasad Eye Institute

Road #2, Banjara Hills

Hyderabad – 500 034

India

Email: [premnandhini@lvpei.org](mailto:premnandhini@lvpei.org)

**Abstract**

**Purpose:** Acute acquired comitant esotropia (AACE) once considered rare, is now on the rise. Surgical management in these cases can get delayed when the angle of deviation varies or if the patient is unwilling. In such cases optical management and vision therapy can be considered. We present the outcomes of such management for AACE.

**Methods:** This retrospective study included a cohort of patients visiting our institute with non-accommodative or non-neurological AACE from January to October, 2023. Fresnel prisms were given if patients had constant double vision. All patients were given vision therapy exercises. Eso deviation and divergence range was measured before and after the therapy.

**Results:** Fourteen patients were included. Of these, 5 patients (with magnitude of eso deviation 20PD or less) were relieved with only vision therapy exercises. For the remaining 9 patients, prisms were given along with vision therapy. Post-vision therapy, 2 patients could maintain single vision without prisms, and in 4 patients the prism power was reduced. In the remaining 3, full prism correction was warranted, one opted for surgery. The median reduction in eso deviation by 6.5PD (range:2-25PD) and increase in divergence by 7PD (range:0 -17PD) after vision therapy was significant (p<0.03).

**Conclusion:** Non-surgical intervention was helpful in reducing eso deviation in 79% (11/14) of patients in this cohort. Certain types of AACE seem to be amenable to vision therapy and/or optical management. Temporary Fresnel prisms can be considered in these patients and weaned away later, if the divergence range can be improved with vision therapy. **Keywords:** Acquired esotropia, double vision, divergence insufficiency, convergence excess, vision therapy, orthoptics, prisms

**1. Introduction**

Acute acquired comitant esotropia (AACE) presents with double vision usually of sudden onset, with inward deviation. The inward deviation can alternate between either eye. The deviation can be constant or intermittent. The condition can occur in both children and adults.(1, 2) This condition has been considered to be relatively rare.(3) However, in recent times there has been an increased incidence of this condition.(4) Excessive near work, particularly with digital devices is considered as a potential etiological factor for this increase.(4-6) Typically, the management of AACE includes Botulinum toxin injection and surgery.(7, 8) Prisms are also considered for relieving double vision.(9) Few studies have shown prisms can also be weaned gradually.(10, 11)  Similar to the reported trends, we have also observed an increase in cases of AACE in our institute. We found that the number of new cases of AACE reported in 2018 at our referral tertiary eye care institute was about 1 in 1000 and in 5 years this increased to 2 in 1000.

The decision for surgical alignment of the eyes is usually taken by the strabismologist after assessing the deviation on different visits within a time period of up to 6 months. In these visits, the variability (or lack thereof) in the eye deviation is determined. In the interim time, or if the patient is not ready for surgery, they are managed by patching their eye to avoid diplopia or given prisms to aid fusion or botulinum toxin injection to reduce the eso deviation. For prism trial and prescription, patients are cross-referred to the Binocular Vision & Orthoptics clinic within the institute. In this clinic, in addition to prescribing prisms, patients are also given vision therapy exercises (in-office or home based) to improve their divergence range after achieving single binocular vision (with or without prisms). Given that these patients earlier had binocular single vision, therefore training them back to that state, with vision therapy appears possible. It has been observed that eso deviation acquired in spasm of near reflex, can be relieved with optical measures and vision therapy.(12) In a similar vein, in this study we present the outcomes of a non-surgical intervention for AACE patients. The management protocol of using prisms and vision therapy, to relieve diplopia and decrease the eso deviation are also described.

**2. Materials and Methods**

A retrospective study was conducted. The study protocol adhered to the tenets of Declaration of Helsinki and was approved by the institutional review board. Medical records of patients who visited the Binocular Vision & Orthoptics clinic from 01/01/2023 to 31/10/2023 were reviewed. Data collected was anonymized. Patients referred to this clinic with a diagnosis of AACE (made by the pediatric and/or neuro-ophthalmologists) were included. Amidst these patients, the following types of AACE, as per the newer classification,(13) types I (Swan, occlusion-related) and II (Burian-Franceschetti, idiopathic) were included and types III (accommodative), IV (decompensated, post-surgical), V (neurologic), VI (cyclic) and VII (secondary) were excluded. Patient’s history was documented and use of smartphones particularly in supine position was also enquired. This question was asked, since we have observed patients to give such a history of using smartphones in supine position for long durations, especially during the COVID-lockdown time and presenting with eso deviation.(5) All the methods described here are the usual clinical tests followed in this clinic.

**2.1 Binocular Vision Assessment**

All patients underwent cycloplegic refraction, prior to the binocular vision assessment. Binocular vision assessment was performed with the best refractive correction. The measurements include: stereoacuity for near with Randot stereo test (Stereo Optical Company, Inc.), qualitative measures of fusion with Worth-four dot test at 40 cm and red lens test over one eye for fusion check at distance. The red lens test has lower dissociation when compared to the Worth-four dot test. In this test, the red lens is placed over one eye, and the patient is asked to report the number of charts seen and the color for the same. In the presence of diplopia, a white and a red chart would be seen distinctly separately. If fusion is present, the patient would report seeing one chart with a light red tinge. Prism cover test was performed for distance and near. If the eso deviation for distance and near were comparable within 4PD (prism diopter), it was termed basic eso deviation. If the eso deviation was greater for distance than near, it was termed divergence insufficiency, vice-versa was convergence excess.

Subjective measures of eye deviation were also made with the Vision Therapy System version 4 (VTS 4, HTS inc., Gold Canyon, AZ, USA). The VTS4 has an integrated 3D screen with stereo goggles that allows dichoptic presentation of the stimuli.(14) The “phoria” module in VTS4 was used, where the patient was asked to align the plus to the immovable circle, using buttons on an X-box (Figure 1a). The testing distance was set at 84 cm, and the patient wore stereo-goggles. The angle of deviation was the distance between the plus and the circle, and this was displayed in prism diopters. While the prism cover test gives the objective measure, with VTS4 the subjective angle of deviation can be measured. With VTS4, magnitudes beyond that measured with Muscle Imbalance Measure/Thorington card, and simultaneous measure of both horizonal and vertical deviation can be achieved.

Vergence ranges (convergence & divergence) were also measured using the VTS4 system, if the patient could maintain fusion at the testing distance. In this test, dichoptic targets of a dolphin with lines around it on 4 sides (up, down, left and right) were shown. The patient was asked to press the side of the “odd one”. While on 3 sides the lines would appear as plus, only on one side (odd one) it will not (Figure 1b). If the odd one is detected correctly, the vergence demand was increased automatically till the patient reported seeing two dolphins, at which point, the judgment of the odd one was also difficult. This point is taken as the break point. The vergence demand was then automatically decreased, till the patient regained fusion to detect the odd one. This point was taken as the recovery point. Besides vergence, accommodation was also measured to ascertain accuracy with dynamic retinoscopy (monocular estimation method, MEM). Other measures of accommodation such as amplitude of accommodation and accommodative facility were also documented.

**2.2 Prism correction**

Fresnel prisms were prescribed to alleviate constant diplopia. The magnitude of the least prism diopter that aided fusion for distance and near was prescribed. Typically, the Fresnel prism is dispensed only over one eye as it hampers contrast and reduces visual acuity. However, we modified this protocol. The prism was split equally between the two eyes, so that the same Fresnel sheet can be cut into two and stuck on each spectacle lens. For example, if 30PD base out eliminated the diplopia, then a 15PD Fresnel sheet was dispensed, and each eye was given 15PD base out. Splitting the Fresnel prism between the two eyes was economical for the patient and easy for dispensing. Patients were given vision therapy for divergence exercises while wearing the prisms. When the patient improves in their divergence amplitude with vision therapy, one of the Fresnel sheets can be peeled away, to reduce the prism magnitude by half. With the reduced prism, the patient is again given the divergence exercises. In the subsequent visits, a decision can be made to either remove the other prism completely or to reduce the prism magnitude further.

**Figure 1:** VTS4 system that shows a stereo TV with dichoptic targets that will be seen using stereo-goggles. (a) phoria measurement, where the person needs to align the blue cross into the red circle with an X-box. The inset shows the magnified view of the dichoptic targets. (b) Flat fusional range measurement task. Through the dichoptic stereo-goggles, only one dolphin would be seen when fused, with lines around it. The patient is asked to identify the side of the odd one by looking at the lines. In this image, the odd one is down. In the remaining 3 sides, a red plus will be seen with fusion.

**2.3 Vision Therapy Protocol**

The battery of divergence exercises for in-office therapy includes double aperture ruler, vectogram, VTS4 divergence exercises given at different viewing distances (84cm, 2m, 3m), accommodation exercises (included due to the coupling between accommodation and vergence system), and Brock string. Patients were also encouraged to do the Brock string exercise at home. For those who could not come for in-office therapy only Brock string exercise was given for a minimum duration of 20 minutes every day, twice a day. The typical in-office vision therapy was given for a duration of 45-60 minutes in a session.

With Brock string, the patients were oriented to appreciate physiological diplopia. They were asked to maintain the fixated bead single for 20 seconds and encouraged to move that bead farther away till the threshold for double is reached. At this break point, they were encouraged to see if they can make the bead single again. When not possible, the bead was brought in a little closer to achieve single vision, and again from that distance, the patient was encouraged to take the bead back, maintaining clear, single vision. Patients were instructed to monitor the break point distance and to increase that distance every day. With Brock string, vergence rock exercises were also combined, where the patients were asked to look at the 3 beads at different distances in the string, each time to hold the fixated bead clear and single for about 20s, while appreciating physiological diplopia. Such an exercise would improve the vergence facility to make eye movements without noticing the double vision and locking fusion seamlessly at different viewing distances. For in-office therapy, usually 10 sittings that can be completed in 2-weeks or spread over few weeks (depending on patient’s ability to come) were given. Binocular vision parameters were measured before (baseline) and after the vision therapy sessions.
**2.4 Data Analysis**

The primary outcome parameters to assess improvement were eso deviation measured before and after therapy (with and without prism spectacles), magnitude of negative fusional vergence (or divergence amplitude) before and after therapy. All the measurements were made for both distance (3m) and near (40cm). Non-parametric test for paired comparison (Wilcoxon Signed Ranks test) was done in SPSS version 21 (IBM Corporation) due to the non-normality of the data (Kolmogorov-Smirnov test, P<0.05).

**3. Results**

Thirty-four patients with diagnosis of AACE visited the Binocular Vision and Orthoptics clinic in the given time period of 10 months. Table 1 shows the demographic details of all these patients. There were significantly (χ^2^=4.2, df=1, p=0.04) more males (n=23, 68%). Of the 34, 14 patients completed the vision therapy (in-office and/or home vision therapy) exercises and came for follow up. Others did not meet the inclusion criteria, had or opted for surgery, did not take vision therapy or were lost to follow-up. Only the results of the 14 patients (64% males) who completed the therapy are discussed further (Table 2).

**Table 1:** Demographics of all the patients (n=34), along with the sub-group of patients (n=14) who were able to complete the vision therapy

|  | 34 patients | 14 patients |
| --- | --- | --- |
| Mean Age± standard deviation (SD) (years) | 24±9.9, Range: 8 to 53 | 22±8.8, Range 10 to 37 |
| Males: Females (n) | 23:11 | 9:5 |
| Digital Device (DD) use (hours/day), Mean ± SD | 8±3.96, Range: 1-14 | 7±3.93, Range: 1-14 |
| DD use in supine position yes: no: not available (n) | 19:3:12 | 7:2:5 |
| Duration of onset of the condition (months) | Range: 2 to 96 | Range: 4 to 48 |
| Distance eso deviation (prism diopters), Mean ± SD | 27± 14,  Range: 8 to 55 | 28±12,  Range: 12 to 45 |
| Basic eso deviation (n) | 23 | 9 |
| Divergence Insufficiency (n) | 8 | 3 |
| Convergence excess (n) | 3 | 2 |

Of the 14 patients, 5 (36%) did not require any prism correction. The eso deviation for distance in these patients ranged from 12 to 20PD, and these patients only had intermittent not constant diplopia. In the remaining 9 patients, for 8, temporary Fresnel prism was dispensed to manage their constant diplopia, and one patient wanted ground prisms for cosmetic reasons. The eso deviation for distance in these patients were ≥20PD. After vision therapy, in 6 patients the prescribed Fresnel prism magnitude could be reduced (n=4) or removed (n=2) with therapy. No patient complained of diplopia after the therapy and or with prisms. The median reduction in eso deviation for all patients at the end of therapy (without the prism correction if any) was 6.5PD (range: 2 to 25PD) for distance and 6PD (range: 0 to 29PD) for near. Both these reductions were significant (Wilcoxon signed rank, p<0.009). Figure 2 shows the distribution of the eso deviation measured with cover test for both distance and near, with and without the prism prescription (if any).

The average duration of digital device use was 7.1±3.9 hours in our cohort. Seven out of 9 patients reported having the habit of using the smartphone in supine position, while the other 2 did not. This history was not documented in the remaining 5 patients. 9 patients underwent in-office vision therapy, and 5 patients took home vision therapy.

Negative fusional range (divergence) was measured with the prism correction (if any). This is because the patient should be free of double vision to be able to perform this test. Figure 3 shows the negative fusional vergence range for distance in 11 patients. Data was not available for others. A statistically significant improvement in the divergence was found for both distance (Wilcoxon signed rank, Z=-2.2, p=0.027) and near (Wilcoxon signed rank, Z=-1.9, p=0.046) after vision therapy. Median improvement of divergence was 7PD (range: 0 to 17PD) for distance and 8PD (range: 0 to 14PD) for near. At the end of therapy, one patient (case 11, in Table 2) did not show any improvement in divergence or reduction in eso deviation. This patient opted for surgical management.

**Figure 2:** Boxplot of the cover test values pre- and post-vision therapy (VT) for both distance (D) and near (N) for 12 patients. Cover test values with and without prisms are also shown.

**Table 2:** Clinical profile of the 14 patients included in the study, before and after therapy (prism and/or vision therapy)

| S.No | Age (years)/  Gender | SE (DS)  RE/LE | Cover test (PD)  Distance/Near | | | NFV (PD)  Distance | | Prism Rx (PD) | | Duration (Months)/ mode of therapy |
| --- | --- | --- | --- | --- | --- | --- | --- | --- | --- | --- |
|  |  |  | Pre | Post   (with prism) | Post (without prism) | Pre | Post | Pre | Post |  |
| 1 | 13/M | +1.00/  +1.00 | 12/10 | 8/0 | 8/0 | - | - | - | - | 3-6/HVT |
| 2 | 23/F | -4.50/ -4.00 | 16/ 12 | NR | NR | 0 | 4 | - | - | <1 /IVT |
| 3 | 10/M | -5.50/ -3.50 | 16/16 | 14/ 16 | 14/ 16 | 1 | 18 | - | - | <1 & 1-3/IVT & HVT |
| 4 | 36/M | 0.00/  0.00 | 18/ 20 | 0/0 | 0/0 | 4 | 12 | - | - | <1/IVT |
| 5 | 23/M | -3.25/ -2.75 | 20/ 20 | 18/18 | 18/18 | 0 | 0 | - | - | <1/IVT |
| 6 | 32/M | -1.50/ -1.25 | 20/8 | 0/0 | 8/8 | 0 | 6 | 8 | 8 | 1-3/HVT |
| 7 | 37/M | -1.50/ -1.50 | 25/20 | 8/6 | 8/6 | 0 | 7 | 16 | 0 | <1/IVT |
| 8 | 24/F | -2.00/  0.00 | 25/16 | 20/12 | 20/12 | - | - | 10 | 0 | 3-6/HVT |
| 9 | 15/M | -7.75/ -7.75 | 35/40 | 12/ 20 | 22/10 | 0 | 4 | 20 | 10 | <1& 1-3/IVT& HVT |
| 10 | 25/M | -4.00/ -4.00 | 40/40 | NR | NR | 0 | - | 24 | 12 | <1/IVT |
| 11 | 28/F | -4.50/ -5.50 | 40/40 | 2/3 | 32/33 | 0 | 6 | 30 | 30 | 1-3 & 3-6/IVT & HVT |
| 12 | 15/F | -5.50/ -5.75 | 45/ 45 | 30/30 | 40/40 | 0 | 1 | 20 | 10 | <1/IVT |
| 13 | 19/M | 0.00/  0.00 | 45/ 45 | 10/6 | 20/16 | 0 | 7 | 35 | 10 | >6/HVT |
| 14 | 11/F | +1.50/  +1.25 | 45/55 | 0/10 | 30/40 | 0 | 7 | 30 | 30 | 1-3/HVT |
| SE: spherical equivalent of the refractive error, DS: diopter sphere, RE: right eye, LE: left eye, PD: prism diopters, M: male, F: female, HVT: home vision therapy, IVT: in-office vision therapy, NR: not recorded. | | | | | | | | | | |

Distance NFV (Prism Diopters)

|  | 2 | 3 | 4 | 5 | 6 | 7 | 9 | 11 | 12 | 13 | 14 | Average |
| --- | --- | --- | --- | --- | --- | --- | --- | --- | --- | --- | --- | --- |
| Pre | 0 | 1 | 4 | 0 | 0 | 4 | 0 | 0 | 0 | 0 | 0 | 0.818182 |
| Post | 4 | 18 | 12 | 0 | 6 | 7 | 4 | 8 | 11 | 7 | 7 | 7.63636 |

**Figure 3:** Negative fusional vergence (NFV) range for distance, measured pre- and post-vision therapy for each individual subjects (grey lines). The thick black line represents the average NFV. Overall, an improvement in NFV values is seen.

**4. Discussion**

This retrospective study investigated the outcomes from non-surgical management of AACE with prisms and/or vision therapy in 14 patients. Eso deviation and divergence range were the outcome measures after the management. We observed that none of the patients experienced double vision with prisms and/or vision therapy. Eso deviation of 20PD or less was managed with vision therapy alone to achieve stable single vision (n=5). In those patients requiring prisms (64%, n=9), single vision was achieved with prism power lower than the eso deviation magnitude and the prism power was reduced (n=4) or removed (n=2) with vision therapy exercises in some of these patients (Table 2). Only in 3 patients (21%) the magnitude of the initial prism power did not reduce.

Reduction in eso deviation and improvement in divergence was also observed in this study (Figures 3 and 4). Synchronously, in both these parameters the median reduction for eso (6.5PD) and improvement for divergence (7PD) was comparable. While evidence for the effectiveness of vision therapy is well established for convergence insufficiency,(15) it is not the case for divergence. It is believed that the first line of management for divergence insufficiency is to prescribe prisms.(16) The results of this study show that divergence can also be improved with vision therapy exercises. Divergence therapy can be facilitated with the help of prisms, to first achieve single binocular vision, if the diplopia is constant, after which divergence can be trained. Majority of the patients in this cohort (68%) had basic eso deviation, followed by divergence insufficiency (25%). Unlike exo deviation that is more bothersome for near work, with eso deviation, the distance double vision is more bothersome. This may trigger the patient to avoid distance tasks and do more near work, which could then further vergence adapt them into the eso posture. Hence, relieving them of the double vision for distance, with advice to minimize near work or increase the near working distance (i.e. move objects further away) is a practical solution.

In a recent study(11) prisms were prescribed and weaned for eso deviation of 25PD or lower in patients with AACE. In our study we observed that prisms were mostly not needed if the deviation was 20PD or less. Improvement was obtained with only vision therapy exercises. Additionally, it was also found through this study that larger deviations (>25PD) can also be managed with a combination of both prisms and vision therapy exercises. However, a residual eso deviation remained in these patients. In recalcitrant cases where the eso deviation is not reduced, surgical management can be considered.

The Fresnel prism was split between the two eyes, to make it both economical and easier to wean the prism. Bilateral Fresnel prism was considered in an earlier study(9). However, this study did not indicate about weaning the prisms. In general, the blur induced by the Fresnel prism may not be tolerated by patients. However, given the temporary nature of these prisms, and that it was expected to get weaned away, our patients did not mind the blur. For those patients who had the need to use the residual prism power, the final Fresnel prism power was given only for one eye. So that the other eye (usually dominant eye) can be kept blur free. This could help promote tolerance to the Fresnel prism spectacles. Else, ground prisms can also be considered for better acceptance (both for cosmesis and clarity). One patient (case 6, Table 2) in this study took the ground prism spectacles.

The skewed gender ratio observed in this study has not been highlighted before. In a recent study on AACE(17), 65% of the patients were males, a percentage comparable to our study (68% males). The reason for this gender predisposition is unclear. It has been observed that adult females tend to practice the 20-20-20 rule more than males. (18) However, the history on practice of the 20-20-20 rule was not documented in this study. Like earlier studies, (9, 11, 17) majority (71%) patients had myopic refractive error (Table 2) and could perhaps be having a Bielschowsky type of AACE. (13)

Even though several precipitating etiologies have been described,(13) the exact pathogenesis of AACE is unclear. Vergence adaptation is said to occur when a prolonged vergence posture is maintained. This could be possible either by wearing a prism for a prolonged time or by viewing at a near distance for a prolonged time.(19, 20) The dynamics of vergence eye movements also changes when vergence adaptation sets in. (21, 22) Hence, prolonged near work could result in vergence adaptation, and this could be abnormal in individuals with binocular vision anomalies.(23) Such an adaptation could reset the baseline vergence status of an individual. Such a mechanism could be considered similar to that of accommodative spasm that could be triggered with close work. (24) Spasm of near reflex (SNR) involves both accommodation and vergence of the near triad. SNR with only the accommodative component has been found to be more common than its vergence counterpart. (12) It is possible that the vergence component presents as AACE. The underlying mechanism of SNR spectrum could have a shared etiopathogenesis, that could also involve AACE. This speculation needs further careful examination.

The average duration of the smartphone or digital device use in this cohort seems to be comparable to the general population. (18) The viewing distance for the digital device use was not documented in these patients. It is known that a closer viewing distance could increase the asthenopic symptoms.(25) While the question of smart phone use in supine position was asked, it is not known how many in the general population adapt such a posture while using their phones. Hence a concrete argument cannot be made that the supine position triggers AACE. A small but real effect has been found in ocular parameters in different head postures.(26) While these experimental studies were done for relatively shorter duration, in real world scenario, the digital device use duration could run into hours. Hence the effect could be even greater with the complex interplay through the cross-links of AC/A and CA/C. All this needs further investigation.

The retrospective nature of this study does pose some limitations. The history could have been more curated, perhaps with a checklist. There were some missing data, but we do not think the overall trend would have been affected. Given that the patients came for different therapy durations or with different modality of therapies (home vs office-based vision therapy), some heterogeneity and variability can be present. Nevertheless, the overall trends of decrease in eso deviation through objective measures, and improvement in divergence are all statistically significant and clinically meaningful as well. There was no control subjects included in this retrospective study. Ideally, this would have been patients who did not take prisms and/or vision therapy but observed for the natural course of the condition. However, given the duration of onset in the included patients ranged from few months to more than 1 year (Table 1), it is evident that the condition can be long standing and yet be amenable to the management protocol described here. This is the first study to show an effective management in some types of AACE using vision therapy exercises with or without prisms.

In conclusion, non-accommodative, non-neurologic types of AACE can be managed non-surgically through a combination of prisms and/or vision therapy in 79% of patients. Specifically, divergence range can be improved and eso deviation can be decreased with this management technique. Therefore, vision therapy with or without prisms can also be considered for managing AACE.

**Conflict of Interest:** None

**Acknowledgment:** Hyderabad Eye Research Foundation for their support to carry out the project.

**References**

1.	Clark AC, Nelson LB, Simon JW, Wagner R, Rubin SE. Acute acquired comitant esotropia. Br J Ophthalmol. 1989;73(8):636-8.

2.	Simon AL, Borchert M. Etiology and prognosis of acute, late-onset esotropia. Opthalmology. 1997;104(8):1348-52.

3.	Roda M, di Geronimo N, Valsecchi N, Gardini L, Fresina M, Vagge A, et al. Epidemiology, clinical features, and surgical outcomes of acute acquired concomitant esotropia associated with myopia. PloS one. 2023;18(5):e0280968.

4.	Zhu M, Tang Y, Wang Z, Shen T, Qiu X, Yan J, et al. Clinical characteristics and risk factors of acute acquired concomitant esotropia in last 5 years: a retrospective case–control study. Eye (London, England). 2023;37(2):320-4.

5.	Vagge A, Giannaccare G, Scarinci F, Cacciamani A, Pellegrini M, Bernabei F, et al. Acute Acquired Concomitant Esotropia From Excessive Application of Near Vision During the COVID-19 Lockdown. J Pediatr Ophthalmol Strabismus. 2020;57(6):e88-e91.

6.	Lee HS, Park SW, Heo H. Acute acquired comitant esotropia related to excessive Smartphone use. BMC Ophthalmol. 2016;16:37.

7.	Lang LJ, Zhu Y, Li ZG, Zheng GY, Peng HY, Rong JB, et al. Comparison of botulinum toxin with surgery for the treatment of acute acquired comitant esotropia and its clinical characteristics. Sci Rep. 2019;9(1):13869.

8.	Sheth J, Goyal A, Natarajan D, Warkad VU, Sachdeva V, Kekunnaya R. Clinical Profile, Neuroimaging Characteristics, and Surgical Outcomes of Patients With Acute Acquired Non-accommodative Comitant Esotropia. J Pediatr Ophthalmol Strabismus. 2023;60(3):218-25.

9.	Chang F, Wang T, Yu J, Li M, Lu N, Chen X. Prism treatment of acute acquired concomitant esotropia precipitated by visual confusion. Strabismus. 2020;28(1):7-12.

10.	Wu Y, Feng XL, Chang M, Gao XQ, Li JH, Kong M. Short-term outcomes of prism treatment in a manner of reducing prism diopters step by step for small-angle acute acquired concomitant esotropia. Zhonghua Yan Ke Za Zhi. 2022;58(3):187-93.

11.	Wu Y, Feng X, Li J, Chang M, Wang J, Yan H. Prismatic treatment of acute acquired concomitant esotropia of 25 prism diopters or less. BMC Ophthalmol. 2022;22(1):276.

12.	Roy S, Bharadwaj SR, Patil-Chhablani P, Satgunam P. Spasm of near reflex: a comprehensive management protocol and treatment outcomes. Journal of American Association for Pediatric Ophthalmology and Strabismus. 2021;25(3):162 e1- e6.

13.	Buch H, Vinding T. Acute acquired comitant esotropia of childhood: a classification based on 48 children. Acta ophthalmologica. 2015;93(6):568-74.

14.	Sehgal S, Satgunam P. Quantifying Suppression in Anisometropic Amblyopia With VTS4 (Vision Therapy System 4). Translational vision science & technology. 2020;9(12):24.

15.	CITT, C.I.T.T.S.G. Randomized clinical trial of treatments for symptomatic convergence insufficiency in children. Archives of ophthalmology (Chicago, Ill : 1960). 2008;126(10):1336-49.

16.	Scheiman M, Wick B. Clinical Management of Binocular Vision: Heterophoric, Accommodative, and Eye Movement Disorders, Philadelphia, PA: J. B. Lippincott Co. 1994:262.

17.	Meng Y, Hu X, Huang X, Zhao Y, Ye M, Yi B, et al. Clinical characteristics and aetiology of acute acquired comitant esotropia. Clinical & experimental optometry. 2022;105(3):293-7.

18.	Datta S, Sehgal S, Bhattacharya B, Satgunam P. The 20/20/20 rule: Practicing pattern and associations with asthenopic symptoms. Indian J Ophthalmol. 2023;71(5):2071-5.

19.	Ehrlich DL. Near vision stress: vergence adaptation and accommodative fatigue. Ophthalmic & physiological optics : the journal of the British College of Ophthalmic Opticians (Optometrists). 1987;7(4):353-7.

20.	Henson DB, North R. Adaptation to prism-induced heterophoria. American journal of optometry and physiological optics. 1980;57(3):129-37.

21.	Satgunam P, Gowrisankaran S, Fogt N. The influence of vergence adaptation on open-loop vergence dynamics. Vision research. 2009;49(14):1795-804.

22.	Lee YY, Granger-Donetti B, Chang C, Alvarez TL. Sustained convergence induced changes in phoria and divergence dynamics. Vision research. 2009;49(24):2960-72.

23.	North R, Henson DB. Adaptation to prism-induced heterophoria in subjects with abnormal binocular vision or asthenopia. American journal of optometry and physiological optics. 1981;58(9):746-52.

24.	Satgunam P. Relieving Accommodative Spasm: Two Case Reports. Optometry & Visual Performance. 2018;6(5).

25.	Ramteke S, Satgunam P. At what distance should digital devices be viewed? Eye (London, England). 2024;38(4):815-6.

26.	Atchison DA, Claydon CA, Irwin SE. Amplitude of accommodation for different head positions and different directions of eye gaze. Optom Vis Sci. 1994;71(5):339-45.
