# Supplementary material for: Management of acute acquired comitant esotropia using prisms and vision therapy
Source: PLoS One. 2026 Feb 23;21(2):e0340010. doi: 10.1371/journal.pone.0340010 (PMC12928452; doi:10.1371/journal.pone.0340010)
Supplement: Attachment — Submitted filename: Response to Reviewers_21Jul2025.docx [file pone.0340010.s004.docx]

We thank the Editors, Reviewers and Journal Office for their time and comments. We have now addressed these comments as appropriate. The reply is included below in blue. **All the changes are highlighted or tracked in the manuscript** and the changes made, along with the line numbers, are indicated in the reply below. A clean version of the manuscript is also added. We have now formatted the paper as per PLOS ONE guidelines. We have also clarified the funding information in the cover letter now.

Additional Editor Comments:

The manuscript could benefit from English editing, with some minor suggestions below.
Additional comments specified below include lack of references, lacking details in the methods section, and ambiguity in Figure 3

Introduction
Lines 2,3- require references

We have now included two references (reference 1 & 2), (line 2 changes are highlighted).

Line 5- however in recent years, its incidence has been on the rise

Thank you. Changed as suggested, highlighted in the manuscript (lines 4-5)

Line 16- in the interim (without time)

Thank you. Changed as suggested. Deletion is tracked (line 15)

Line 17- remove “their eye”

Thank you. Changed as suggested. Deletion is tracked (line 16)

Line 23- previously demonstrated binocular single vision, training with vision therapy to revert to the prior condition, renders vision therapy plausible.

Thank you. Changed as suggested. Changes are highlighted (lines 22-23)

Line 35- amongst these patients

Thank you. Changed as suggested. Change is highlighted (line 34).

Line 42- The methods described herein are the typical clinical tests conducted in this clinic.

Thank you. Changed as suggested. Changes are highlighted (line 43).

Line 45- which drug? And how many drops per eye?

Thank you for this question. The following details are now added: one drop of 1% cyclopentolate hydrochloride and one drop of tropicamide plus (0.8% tropicamide +5% phenylephrine hydrochloride). Changes are highlighted (lines 46-47).

Line 49- to examine distance fusion

Thank you. Changed as suggested. Changes are highlighted (line 51).

Line 50- provide a references that the red lens test has a lower dissociation compared to the Worth four dot test.

Thank you. We have now included the needed reference (reference number 14). Change is highlighted (line 52).

Line 51- the red lens test is typically performed with a penlight or spot of light. It is unclear why the patient would be expected to report several charts

In this test, the red lens is placed over one eye and patient is asked to view a computer monitor (computer chart in our case) to appreciate the diplopia if any in room light condition. This test mimics a real-world setting more closely than the typical penlight and red filter test done in dark room. We have now added this additional information (lines 53-55).

Line 53: red tint?

We believe tinge is also acceptable. It denotes the visual perception of the person.

Lines 55-57: provide a reference for this (relationship between phoria at distance and near to determine the type of impairment)

We have now included the needed reference (reference number 15). Change is highlighted (line 61).

Line 83: provide a reference for Fresnel prism hampering contrast and reducing visual acuity

We have now included the needed reference (reference number 17). Change is highlighted (line 88).

Line 88: why is dispensing a Fresnel sticker for both eyes more economical that just one eye? Doesn’t it cost double the amount?

We dispense only one sheet that is cut for the two eyes and therefore the patient pays only once. With therapy one of the sheets can be removed (essentially halving the prism), when improvement is observed. We now explicitly say ‘one’ Fresnel sheet and have also added additional details to improve clarity. Changes are highlighted (lines 91-92).

Figure 1: below instead of down

Thank you. Changed as suggested. Change is highlighted.

Line 96: I believe it is an aperture rule and not ruler

Thank you for this correction. This is fixed now (line 101).

Line 100: for a specific time at home? Or everyone does however much they want?

Thanks for this question. They are instructed to do for 10-20 minutes per day. Change is highlighted (line 106).

Line 100: those who did not come and were given Brock String 20 min per day twice a day- were they given a tutorial? How did they know what to do with the Brock string?

Yes, they were given an orientation to this exercise during their clinic visit. We have now added this information (lines 108-109).

Line 123 – performed

Thank you. Changed as suggested. Change is highlighted (line 131).

Line 127: Table 1 displays, delete the word “all”

Thank you. Changed as suggested. Changes are highlighted and tracked (line 135).

Line 130: arrived to their follow-up appointment

We have now modified this phrase as came for “their” follow up visit. Changes are highlighted (line 138).

Line 131: did not participate in vision therapy

Thank you. This is now changed as did not “undergo” vision therapy. Change is highlighted (line 139).

Line 135: five (spell out numbers lower than 10)

Thank you. Changed as suggested in this line (line 143) and elsewhere as well. All changes are highlighted.

Line 138: preferred instead of wanted

Thank you. Changed as suggested. Change is highlighted (line 146).

Line 141: remove “with therapy” as you wrote “After vision therapy” in the beginning of the sentence

Thank you. Removed as suggested. Deletion is tracked (line 149).

Line 149: two and five, spell out nine

Thank you. Changed as suggested. Changes are highlighted (lines 155-158).

Figure 3: Is this the break point? When you say “range”- do you mean the average of break and recovery? Not clear..

Thank you for this question. It is indeed the break point. We have now changed this in the figure legend. Change is highlighted.

Line 158: three

Thank you. Changed as suggested. Change is highlighted (line 178).

Line 178: The majority

Thank you. Changed as suggested. The change is highlighted (line 188).

Line 229: was found

Thank you. Changed as suggested (line 239).

Line 243: ranging from

Thank you. Changed as suggested (line 253).


Reviewer #1:

This is a well-written and insightful article. It highlights the importance of promoting non-surgical management approaches in similar cases. Articles like this not only enhance clinical understanding but also encourage practitioners to explore and implement conservative, evidence-based treatment options where appropriate.

We thank the reviewer for their positive comments and encouragement.

Reviewer #2: This manuscript presents a valuable retrospective study examining non-surgical management of acute acquired comitant esotropia (AACE) using prisms and vision therapy. The authors report promising results, with 79% of patients showing improvement through this approach.
The paper is well-structured with a clear rationale, detailed methodology, and meaningful results.
Overall, this study provides clinically relevant evidence for an alternative management strategy for AACE and represents a worthwhile contribution to the field. I suggest some minor revisions.

We thank the reviewer for their positive comments and encouragement.

- Sample Size and Study Design: Address the limitations of the small sample size (n=14) and retrospective design more critically. Consider discussing whether the findings should be considered preliminary until validated in a larger prospective study.

We thank the reviewer for this suggestion. We have now added the following line in the discussion: “These preliminary findings can help plan a larger prospective study.” (line 257).

- Patient Selection Guidelines: a clearer clinical guidelines regarding which AACE patients are suitable candidates for non-surgical management versus those who should proceed directly to surgery would significantly enhance the practical application of your findings.

We thank the reviewer for this suggestion. We have now added the following lines in the Conclusion section:
For most patients with less than 20 prism diopter intermittent eso deviation, vision therapy alone will suffice. For higher magnitudes of deviation along with constant eso deviaiton, prisms can be prescribed to achieve single vision and later can be weaned with vision therapy exercises (lines 262-265).

- Long-term Outcomes: if possibile provide data on the longest follow-up period available and discuss whether the improvements observed were maintained over time.

We thank the reviewer for this suggestion. We have now added the following statement:

The longest follow-up was for 1 year (case 13) and the patient maintained their improved binocularity with Brock string exercise and prism spectacles (lines 167-169)

Reviewer #3: 1. Only patients with esotropia deviation and divergence range outcome measures pre and post management should be included in this manuscript (patients 1,2,8 &10 should be excluded). Hence, table 1, table 2, Fig 2 and Fig 3 should be all, accordingly, reedited.

Thank you for this comment. While we agree that only patients with all values recorded should be included, the retrospective study design unfortunately has some limitations and missing values is one of them. Nevertheless, we have included all patients into the table, essentially to understand the demographics and the presenting clinical profile for these patients. We now mention this as a preliminary study that can help plan future prospective studies (line 257).

Figure 2 discards the two patients on whom the deviation was not measured (indicated in figure legend). Similarly, Figure 3 is plotted only when both pre- and post-data are available. All the relevant statistics were done only for patients with pre and post-measurements.

2. It is advisable to add NFV (PD) at near data, if available. It might contribute for understanding the mechanisms of AACE and treatment success.

Thank you for this suggestion. We have now added these values (when available) into Table 2.

3. The classification mentioned in line 35 is based on research in pediatric population (reference 13). The population of the current manuscript includes adults. The authors should clarify it.

We thank the reviewer for this comment. We now have added the following sentence to clarify the same:

While this classification was developed from a study done on children, we used this classification in our study that included both adults and children. (lines 37-39)

4. Line 53, was it simultaneous prism cover test or alternate cover test?

Thank you for this question. It is alternate prism cover test. We have now clarified this in the text (line 57).

5. The VTS 4 is described, however, it is not clear which measurements with the VTS 4 and at what specific distance were included (for example, measuring subjective eye deviation at the mentioned testing distance of 84 cm is probably irrelevant). Furthermore, the gold standard for measuring NFV at distance and near is with prism bar (was it done?). Was the VTS4 validated for measuring NFV or deviation in children\adults?

Thank you for this important question. The prism bar was used for measuring NFV at distance and near (we have included this information now lines 72-73).

Vergence range was also measured using VTS4. While the values measured between the two (VTS4 and near prism bar test) may be correlated it will not be comparable, since the targets and the testing conditions are different. The normative range for VTS4 is given in the user manual. However, there are no validation studies done using VTS4 and prism bar. Hence, any improvement in vergence range can only be compared against the same tool, and not interchangeably.

6. The subgroup of patients who were able to complete the vision therapy (table 1 first line) were all in the age of pre-presbyopia and had a shorter duration of onset of the condition range. It is worth a discussion.

This is an interesting observation made by the reviewer. Although the ones who completed the vision therapy also include children. So not all were in the age of pre-presbyopia. While it is tempting to speculate on the change in accommodation-vergence relation in pre-presbyopia potentially having a role in AACE, the smaller sample size forces us to apply caution. Therefore, we will refrain from this discussion. Thank you.

7. The authors should clarify the way they measured the post cover test with prisms in glasses. Using two horizontal stacked prisms can induce a measurement error (Thompson JT, Guyton DL. Ophthalmic prisms. Measurement errors and how to minimize them. Ophthalmology. 1983 Mar;90(3):204-10. )

Thank you for this question. Yes, stacking up prisms especially when held in the same direction gives error mainly due to the air gap between prisms. In our patient cohort, only Fresnel prism sheet was used. At the end of therapy, patients had the Fresnel prism worn over one eye only. So neutralizing prisms (plastic prism) can be held over the other eye. We have also observed that holding the prism in front of the eye with Fresnel prism sheet, also gives comparable results.

8. It is advisable to add more detailed vision therapy protocol as an e-supplement .

Thank you for this suggestion, we have added a vision therapy protocol as an e-supplement now.
