# Supplementary material for: Management of acute acquired comitant esotropia using prisms and vision therapy
Source: PLoS One. 2026 Feb 23;21(2):e0340010. doi: 10.1371/journal.pone.0340010 (PMC12928452; doi:10.1371/journal.pone.0340010)
Supplement: Attachment — Submitted filename: Reviewer Comments_15Oct2025.docx [file pone.0340010.s006.docx]

Journal Requirements:

1. If the reviewer comments include a recommendation to cite specific previously published works, please review and evaluate these publications to determine whether they are relevant and should be cited. There is no requirement to cite these works unless the editor has indicated otherwise.

Thank you for this note.

2. Please review your reference list to ensure that it is complete and correct. If you have cited papers that have been retracted, please include the rationale for doing so in the manuscript text, or remove these references and replace them with relevant current references. Any changes to the reference list should be mentioned in the rebuttal letter that accompanies your revised manuscript. If you need to cite a retracted article, indicate the article’s retracted status in the References list and also include a citation and full reference for the retraction notice.

Thanks for this note, there are no retracted articles cited.

Additional Editor Comments :

I commend the authors for their revision. One reviewer is satisfied and the other still requires some changes therefore I recommend a "minor revision".

We thank the reviewers and the Additional Editor for their time to review our revision.

In addition to that reviewer's comments I have three items that I would like the authors to address:
1 The reviewer asked : The authors should clarify the way they measured the post cover test with prisms in glasses. Using two horizontal stacked prisms can induce a measurement error (Thompson
JT, Guyton DL. Ophthalmic prisms. Measurement errors and how to minimize them. Ophthalmology. 1983 Mar;90(3):204-10. )
The authors responded: Thank you for this question. Yes, stacking up prisms especially when held in the same direction gives error mainly due to the air gap between prisms. In our patient cohort, only Fresnel prism sheet was used. At the end of therapy, patients had the Fresnel prism worn over one eye only. So neutralizing prisms (plastic prism) can be held over the other eye. We have also observed that holding the prism in front of the eye with Fresnel prism sheet, also gives comparable results.". I would add this to the discussion for other readers who may have similar questions as they read the manuscript

Thank you for this comment. As recommended, we have now added this information (please see below, in italics) in our manuscript (page 10, lines 156-158). Changes in the manuscript are highlighted.

*While performing the cover test with prism prescription, the neutralizing prism was held over the eye without the Fresnel prism. We also observed that holding the prism in the eye with Fresnel prescription gave similar values.*

2 the authors state that cycloplegia was applied prior to the binocular vision examination, which included stereopsis. Is stereopsis, which is measured at 40 cm and requires 2.5 D of accommodation (approximately) expected to be within normal limits if the patient is cyclopleged? I did not see any stereopsis findings in the tables provided? Also, would any stereopsis be expected in patients with strabismus?

Sorry for this confusion. Binocular vision assessment was made on a separate visit, after the cycloplegic refraction. We have now clarified this in the manuscript (page 5, lines 49-50, changes are highlighted).

In the binocular vision clinic, stereoacuity was tested in undilated conditions. In some of the AACE patients, stereoacuity was present. This is because the near viewing condition facilitates the eso position. Hence those who could maintain bifoveal fixation for near, demonstrated stereopsis. We have now added the stereopsis values observed in Table 2.

3 The authors stated that "If the eso deviation for distance and near were comparable within 4PD (prism diopter), it was termed basic eso deviation." - surely there is a minimal amount that is considered "normal"? If one has 2^ eso at distance and near, is it "basic eso"? Similarly, If the eso deviation was greater for distance than near, it was termed divergence insufficiency, vice-versa was convergence excess. [15]- also here specify the minimal amounts.

As indicated if the difference is within 4PD, it is considered basic type. Except for orthophoria, any deviation is taken into consideration for the classification. So, if one has 2PD eso at distance and near, it is considered as basic eso type. The minimal magnitude may matter depending on the deviation being a phoria or a tropia. For example, upto 6 prism diopters exophoria for near can be considered as normal. However, when the deviation is manifested (constant or intermittent), with patient complaining of double vision, then even a small deviation like 2PD can be abnormal.

Reviewer #4: In this study, the results of prism and/or vision therapy in 14 patients with AACE were reviewed and it was found that the divergence range can be improved and the esodeviation can be decreased with this management technique. In 7 of the 14 cases, prism treatment could be discontinued after prism and/or vision therapy. However, there are also problems:
1、 Of the 14 patients, 5 had variable deviation or intermittent diplopia. Should myasthenia gravis and accommodative spasm be excluded?

Thank you for this question. Yes, patients with accommodative spasm and other systemic diseases, including myasthenia gravis were excluded. We have now clarified this in methods (page 4, line 40, changes are highlighted).

2、 Two classification methods of AACE were mentioned in the study. One was used for inclusion and the other for discussion (line 217). Why is the same literature used for citation? In addition, among the 14 patients, none were under 10 years of age and 11 patients were over 14 years of age. However, the use of the AACE classification for children as the inclusion criteria of the study is still questionable.

Thank you for this observation. In the older classification of 3 types, Bielschowsky type was associated with myopia. In the newer classification as used by Seth et al. (J Ped Ophthal Strab, 2023), Bielschowsky type got included in Type II. We have now clarified this (page 16, line 224, changes are highlighted) and also clarified it in the methods (page 4, line 36, changes are highlighted). We only use the newer classification system in this study (both in methods and discussion).

We agree that the AACE classification was originally described for children. However, this classification has also been used for adults (Seth et al., J Ped Ophthal Strab, 2023).

3、 Table 2 should be supplemented with the classification of AACE, the accommodation measures, the prism diopters worn and the prognosis.

We have included stereoacuity values in Table 2 now. It already has the prism values. Accommodation measures are not included. None of the patients had any accommodative dysfunction or spasm. Prognosis is not included, as this table gives quantifiable measures. All the patients had type II AACE. This information is now added to the results (page 9, lines 140-142, changes are highlighted).

4、 Of the 9 cases treated with prisms, Fresnel prisms were prescribed in 8 cases and group prisms in 1 case. However, only the Fresnel prism was mentioned in the ABSTRACT.

Thank you for this comment. We have now modified the abstract (changes highlighted). In methods the word “Fresnel” is removed. Additionally, the following sentence is also edited in the conclusion of the abstract:

*Temporary Fresnel prisms can be considered in majority of these patients….*

5、 In the 9 patients with a deviation of more than 20PD, prism treatment was first prescribed to eliminate the diplopia. The prisms were evenly distributed to both eyes. What was the prescribed amount of prisms? After the deviation was reduced by vision therapy, the prisms in the non-dominant eye were removed, i.e. the prisms were reduced by half. Were all six patients able to eliminate the diplopia? How is it possible to gradually reduce the prism?

The prescribed amount varied from patient to patient. The amount chosen was based on the minimum prism that eliminated diplopia (lines 87-88). Table 2 shows the prism value prescribed.

Yes, the reviewer is correct in the observation that the prism from one eye was removed, thus reducing the prism value by half. Only in those who were free from diplopia after the removal were left with the remaining (half) magnitude of the prism. We agree that a step jump to half the magnitude cannot be described as “gradual” reduction. We do not report “gradual” reduction in our work. We cite an earlier work that used gradual reduction of prisms (reference #1, Wu et al., 2022).

6、 Are there any results after stopping the vision therapy for three or six months?

In patient ..... longitudinal follow up was possible for xx months.

We had included a note on follow-up for the patient who had a 1-year follow-up (lines 171-173). We now include 3 other patients who followed up within a duration of 2-5 months. All these patients showed a stable binocular vision status (page 11, lines 173-174, changes highlighted).

7、 The treatment results of the five patients who did not wear prisms were not mentioned in the RESULTS section.

The results of the 5 patients who did not wear prism spectacles are included in the Results section (lines 145-147).

8、 In the RESULTS section, it was mentioned that some patients were lost to follow-up. What were the reasons for the loss to follow-up? Is it because the treatment effect is not good?

This is a valid concern. Given the retrospective nature of this study, we are unable to know the reasons for loss to follow-up.

9、 In the DISCUSSION section, it was mentioned that SNR might be a pathogenic factor of AACE. Are there any results of accommodation measures?

We speculate that SNR spectrum could include AACE, if only the vergence component of the near triad is involved. In 12 patients lag of accommodation was documented and in no patients was accommodative spasm observed. We have now included this additional information (page 9, lines 141-142, changes highlighted).

10、 The conclusion mentioned that 79% (11 cases) of patients had a reduction in prism degree after vision therapy. This should include the 5 cases that did not wear prisms, but the deviation of cases 3 and 5 did not decrease significantly.

This number includes the 5 patients without the need for prisms + 6 patients who showed a reduction in prism power. The remaining 3 patients did not show any reduction in their eso deviation or improvement in their divergence amplitude. Cases 3 and 5 showed improvement in their divergence amplitude (Table 1). Although this improvement for Case 5 was minimal.

11、 All 5 cases that did not need to wear prisms achieved the disappearance of diplopia after vision therapy. However, the deviations in 2 cases did not decrease significantly, and the fusion amplitude of case 5 did not increase either. How can it be explained that these two patients were able to eliminate diplopia?

Thanks for this observation. We also noticed that in Table 2, Cover test “Post (with prism)” was irrelevant to these 5 patients, so we have removed those values now, to avoid confusion (changes are highlighted). Cases 3 & 5 showed a reduction in eso deviation by 2PD. The divergence range improvement for Case 3 was much higher when compared to Case 5. Case 3 improvement can be explained by good divergence range improvement. Case 5 on the other hand showed only minimal improvement but was symptomatically better. It’s possible that this patient’s frequency of intermittent double vision could have reduced.

12、 It is recommended to provide photos of ocular alignment before and after treatment for some patients, as this is more intuitive.

Thank you for this recommendation. We have now included the photo of a patient (Fig 4), with informed consent taken from the patient for publication of photo (page 4, lines 32-33, changes highlighted). Informed consent is attached in the supplementary information.
