# Supplementary material for: Management of acute acquired comitant esotropia using prisms and vision therapy
Source: PLoS One. 2026 Feb 23;21(2):e0340010. doi: 10.1371/journal.pone.0340010 (PMC12928452; doi:10.1371/journal.pone.0340010)
Supplement: Attachment — Submitted filename: recommendation-second round.docx [file pone.0340010.s005.docx]

Although the article was revised in the first round, the conclusion remains insufficiently objective and lacks accuracy. The details are as follows:

1. The conclusion should emphasize that prisms and/or vision therapy are intended for patients with variable deviations or for those who are unwilling or not ready to undergo surgery.

2. Prisms and/or vision therapy are helpful for 79% of patients with variable deviations or those not ready for surgery. However, not all of these patients experienced a reduction in deviation. The true clinical value lies in the disappearance of diplopia or its resolution after discontinuing the prism following prisms and/or vision therapy, which occurs in 50% of cases.

3. Among the 9 patients with constant deviations greater than 20 prism diopters, 2 cases (22%) achieved binocular single vision without prisms after treatment. Both of these cases had deviations of 25 prism diopters. This should also be noted in the conclusion.
