# Supplementary material for: Management of acute acquired comitant esotropia using prisms and vision therapy
Source: PLoS One. 2026 Feb 23;21(2):e0340010. doi: 10.1371/journal.pone.0340010 (PMC12928452; doi:10.1371/journal.pone.0340010)
Supplement: Attachment — Submitted filename: Reviewer Comments_4Dec2025.docx [file pone.0340010.s007.docx]

Additional Editor Comments:

We are sorry for the delay, but securing reviewers to re-review took some time.

One reviewer feels that the conclusions should be modified. I think the paper should be read from beginning to end to ensure logical flow. Professional English language editing or reviewing by an English speaking colleague would greatly benefit the final version.

We thank the Additional Editor, for being able to secure a review for the manuscript and for their assistance with this whole review process. We also thank the reviewer for their comments and importantly, for the extensive grammar corrections. We also had our paper reviewed by a native English speaker and a clinician. We have acknowledged her in our paper. The changes made due to language/grammar correction are also tracked.

We invite you to submit a revised version of the manuscript that addresses the points raised during the review process.

Thank you for the opportunity given to revise our manuscript.

Please ensure that your decision is justified on PLOS ONE’s [publication criteria](https://track.editorialmanager.com/CL0/https:%2F%2Fjournals.plos.org%2Fplosone%2Fs%2Fcriteria-for-publication/1/010f019ab97adb9a-6dcc46b9-1516-4d83-a9f8-d8d7003879ff-000000/_AU3Gved4v7dEk9OxjSTl54r9TwBuJeCfuiibmxIgm8=238) and not, for example, on novelty or perceived impact.

Thank you to the authors for revising the submission. I am sorry for the delay- it was difficult to secure re-review for this re-submission.

Thank you for your assistance.

Note that one reviewer that required revisions feels that the conclusion remains insufficiently objective and lacks accuracy. The details are as follows:
1. The conclusion should emphasize that prisms and/or vision therapy are intended for patients with variable deviations or for those who are unwilling or not ready to undergo surgery.

As suggested, we have now included the following lines in the conclusion (lines 289-291, changes are tracked):

*The options of prisms and/or vision therapy can be considered especially for those patients who show variable angles of deviation or who are unwilling or unfit for surgery.*

2. Prisms and/or vision therapy are helpful for 79% of patients with variable deviations or those not ready for surgery. However, not all of these patients experienced a reduction in deviation. The true clinical value lies in the disappearance of diplopia or its resolution after discontinuing the prism following prisms and/or vision therapy, which occurs in 50% of cases.

The observation of 50% of cases (5 who did not need prisms, and 2 who later had the prism removed, so 7 out of 14) is correct. Perhaps these cases can be considered as “treated or resolved”. However, we indicate how many patients were “managed” with prisms or vision therapy (lines 282-283). Along those lines, only in 3 patients (21%) no reduction in prism power was observed, others (79%) either had a reduction in prism power or did not need the prisms. We also indicate in our data analysis that the outcome measures are eso deviation reduction and divergence range improvement (lines 135-137).

3. Among the 9 patients with constant deviations greater than 20 prism diopters, 2 cases (22%) achieved binocular single vision without prisms after treatment. Both of these cases had deviations of 25 prism diopters. This should also be noted in the conclusion.

Thank you for this suggestion. We have now included this information in both conclusions (lines 286-287) and also in discussion (lines 195-197). Changes are tracked.

I would like to add that the authors should read the manuscript from beginning to end to make sure it flows logically.

Thank you for this suggestion. We have done the same. We also had a native English speaker and a clinician to review our manuscript.

For example: I asked the authors how the stereopsis was measured with cycloplegia. They responded that the binocular visual examination was carried out on a separate visit and this was supposedly addressed in the revision. However-
read this section (Lines 47-51):
"All patients underwent cycloplegic refraction (one drop of 1% cyclopentolate hydrochloride and one drop of tropicamide plus (0.8% tropicamide +5% phenylephrine hydrochloride)), prior to the binocular vision assessment. "
Note that it says there was cycloplegia PRIOR (i.e. before) the binocular vision assessment.
Then the authors write in the subsequent sentence " Binocular vision assessment was performed on a separate visit, with the best refractive correction."
This seems to contradict the prior sentence.

Sorry for this confusion. We have now changed that sentence to indicate “..in their initial visit” (lines 50-51, changes are tracked).

Then the subsequent sentence says "The measurements include"- do the authors mean that the binocular vision examination procedures included: (in past tense)?

Yes, it should be in the past tense “included”. We have now corrected the same (line 53), changes are tracked.

The grammar must be fixed here:

Line 52: fusion with the Worth four dot test at 40 cm and a red les test over one eye to examine distance fusion. Add "A" before "red lens"

Thank you. Changed as suggested (line 54, changes are tracked).

Line 56: what does "the color for the same" mean?

We have changed this to “..and the color of the chart” (line 58, changes are tracked)

Line 56: "under normal viewing conditions" instead of "in normal viewing condition"

Thank you. Changed as suggested (line 58, changes are tracked)

Line 60: it was termed a basic eso deviation. Must add "a"

Thank you. Changed as suggested (line 63, changes are tracked)

Line 61: delete the "vice-versa it was convergence excess" and write out the sentence. It makes no sense in its current form.

Thank you. Changed as suggested (lines 64-65, changes are tracked)

Line 71: the Muscle Imbalance - add "the"

Thank you. Changed as suggested (line 74, changes are tracked)

Line 72: can be obtained or measured instead of "achieved"

Thank you. Changed to “obtained” (line 75, changes are tracked)

Line 73: a prism bar- add "a"

Thank you. Changed as suggested (line 76, changes are tracked)

Line 78: instead of "it will not" which is unclear please specify if the authors mean to say that on one side the lines do not appear as a plus?

Thank you. Changed as suggested (lines 81, changes are tracked)

line 78- watch the tenses here. "is detected" should be followed by "is increased" and not "was increased" since later the patient "reported " (past)- perhaps just change everything to past tense? If the odd one WAS detected correctly, the vergence demand was increased automatically until the patient reported seeing two dolphins, at which point the judgment of the odd one was difficult. (delete "also")

Thank you very much for this correction. We have made the necessary changes (lines 81, 83, changes are tracked)

Line 81: until instead of "till"

Thank you. Changed as suggested (line 84, changes are tracked)

Line 91: since you modified the protocol (in the past)- the Fresnel sheet was cut into two

Thank you. Changed as suggested (line 95, changes are tracked)

Line 93: one 15PD Fresnel sheet was cut and dispensed such that each eye received 15PD Base out.

Thank you. Changed as suggested (line 97, changes are tracked)

Line 94: and easy to dispense

Thank you. Changed as suggested (line 99, changes are tracked)

Line 95: when the patient improved

Thank you. Changed as suggested (line 100, changes are tracked)

Line 96: vision therapy, it was possible to peel off one of the Fresnel sheets to reduce the prismatic correction by half.

Thank you. Changed as suggested (lines 100-102, changes are tracked)

Line 97-98: After the prismatic correction was reduced, the patient was dispensed divergence exercises.

This sentence was changed as “With the reduced prism correction, the patient continued the divergence exercises” (lines 102-103, changes are tracked)

Line 98- During subsequent visits, complete removal or reduction of prism magnitude were considered.

Thank you. Changed as suggested (lines 103-105, changes are tracked)

Figure 1 caption: The VTS4 system includes a stere-television with dichoptic targets viewed through stereo-goggles. (a) phoria measurement requiring alignment of the blue cross in the center of the red circle using an XBox. The inset shows the magnified view of the dichoptic targets. (b) ....the patient was asked to identify the odd side. In this image, the odd target is below. In the remaining three slides, a red plus is seen with fusion.

Thank you very much. We have made the suggested changes now (tracked in the figure caption).

Line 102: included

Thank you. Changed as suggested (line 108, changes are tracked)

Line 103: delete the word "given" - just exercises at different viewing distances

Thank you. Changed as suggested (line 109, changes are tracked)

Line 105: systems (plural)

Thank you. Changed as suggested (line 111, changes are tracked)

Line 108: the Brock string exercise

Thank you. Changed as suggested (line 114, changes are tracked)

Line 113: until reaching the diplopia threshold

This sentence is now changes as “…encouraged to move that bead farther away until it was perceived as double” (lines 119-120, changes are tracked)

Line 114: they were encouraged to attempt to fuse the bead into a single bead

Thank you. Changed as suggested (lines 120-121, changes are tracked)

Lines 118-120: Vergence rock exercises were also combined with the Brock String. Patients were asked to look at the three beads at different distances, while maintaining clear and single vision of each bead for approximately 20s, while experiencing physiological diplopia.

Thank you for this suggestion. We have modified this sentence as “Vergence rock exercises were also combined with the Brock string. Patients were asked to look at the beads at three different distances, while maintaining clear and single vision at each fixated bead for about 20s, while appreciating physiological diplopia.” (lines 124-128, changes are tracked).

We chose to retain the word “appreciating” instead of “experiencing”, since we feel that appreciating is a more active process, and that is exactly how we instruct our patients here.

Lines 129-130: All measurements were assessed at distance (3m) and near (40cm).

Thank you for this suggestion. We have modified this sentence as “All measurements were made for both distance (3m) and near (40cm)” (line 137, changes are tracked).

We feel this sentence is also easy to read and conveys the message.

Line 130: A non-parametric test for paired comparisons...

Thank you. Changed as suggested (lines 138-139, changes are tracked)

Line 135: during the study period of 10 months.

Thank you. Changed as suggested (line 143, changes are tracked)

Line 140-141: All of these patients

Thank you. Changed as suggested (line 149, changes are tracked)

Line 147: non-constant

This should say “not constant diplopia”. We have now inserted a comma, to add more clarity to this statement “….only had intermittent, not constant diplopia” (line 156, changes are tracked).

Line 149: was instead of were

Thank you. Changed as suggested (line 159, changes are tracked)

Line 154: Both of these reductions

Thank you. Changed as suggested (line 163, changes are tracked)

Line 162: underwent instead of "took"

Thank you. We retain the word “took”, as suggested by the native English speaker who reviewed this manuscript (line 172)

Line 163: negative fusional range (divergence) was measured with prism correction (if any) to ensure that the patient did not experience diplopia during the testing

Thank you for this suggestion. We have now modified this sentence as “Negative fusional range (divergence) was measured with the prism correction (if any) to ensure the patient did not experience double vision during the test.” (lines 173-175, changes are tracked).

We prefer to use the word “double vision” instead of diplopia, because double vision is the colloquial term for the patient. Also double vision could encompass both diplopia and visual confusion.

Line 185: was not reduced

Thank you. Changed as suggested (lines 197-198, changes are tracked)

Line 186: Not clear which study is "this study"

We have now clarified this to indicate “in the present study” (line 200, changes are tracked)

How about: Additionally, reductions in eso deviation and improvement in divergence were observed.

Thank you. We have changed this sentence as “ A reduction in eso deviation and an improvement in divergence range were observed…” (lines 199-200, changes are tracked)

Line 187: What is the meaning of "Synchronously"- replace
We have removed this word now (line 200, changes are tracked)

Line 209: However, that study did not describe prism weaning.

Thank you. Changed as suggested (line 225, changes are tracked). We are replacing the word ‘weaning’ with ‘tapering’, as suggested by the native English speaker who reviewed our manuscript.

Line 212: it was expected to be reduced

Thank you. Changed as suggested (line 227, changes are tracked)

Line 214: prescribed to only one eye.

Thank you. Changed as suggested (lines 229-230, changes are tracked)

Line 214: This way, the fellow eye (usually the dominant eye) was kept blur-free, promoting tolerance to the Fresnel prism spectacles.

The sentence is changed as “This way the other eye (usually dominant eye) can be kept blur free, promoting tolerance to the Fresnel prism spectacles.” (lines 230-231, changes are tracked)

Line 216: Ground prisms. Delete "else"'

Thank you. Changed as suggested (line 231, changes are tracked)

Line 217: preferred ground prism spectacles.

Thank you. Changed as suggested (line 233, changes are tracked)

Line 246: Previous studies describe short duration experiments, whereas in real-world conditions, digital device may last hours.

We changed the sentence as “While these experimental studies were done for relatively shorter duration, in real world scenario, the digital device use may last hours “ (lines 263-264, changes are tracked)

Line 249 AC/A and CA/C, requiring further investigation.

We prefer to retain the original sentence. Thank you (line 265).

Line 256-257: A control group was not included in this retrospective investigation.

We have changed the “control subjects” to “control group” now (line 273, changes are tracked).

Line 267: ranges
Thank you. Changed as suggested (line 284, changes are tracked)
